# Blood-Based Inflammatory Markers Are Associated with Treatment Outcomes in Head and Neck Squamous Cell Carcinoma Receiving Anti-PD-1 Therapy: CRP as a Superior Predictive Marker

**DOI:** 10.3390/ijms26189154

**Published:** 2025-09-19

**Authors:** Manuel Stöth, Flurin Müller-Diesing, Patricia Mack, Laura Ackermann, Corona Metz, Jan-Peter Grunz, Stephan Hackenberg, Miguel Goncalves, Thomas Gehrke, Till Jasper Meyer, Agmal Scherzad

**Affiliations:** 1 Department of Oto-Rhino-Laryngology, Head and Neck Surgery, University Hospital Würzburg, 97080 Würzburg, Germany; 2 Department of Radiology, Pediatric Radiology, Charité—Universitätsmedizin Berlin, Corporate Member of Freie Universität Berlin and Humboldt-Universität zu Berlin, 10117 Berlin, Germany; 3 Department of Diagnostic and Interventional Radiology, University Hospital Würzburg, 97080 Würzburg, Germany

**Keywords:** HNSCC, immunotherapy, PD-1, biomarker, C-reactive protein, neutrophil-to-lymphocyte ratio, platelet-to-lymphocyte ratio, systemic immune inflammation index

## Abstract

Systemic inflammation is gaining increasing attention as a potential predictive biomarker in immune checkpoint inhibition (ICI) for head and neck squamous cell carcinoma (HNSCC). Several well-established blood-based inflammatory markers are commonly used to estimate systemic inflammatory burden. However, their utility in predicting treatment outcomes in ICI for HNSCC remains unclear. This study aimed to evaluate the predictive value of the following inflammatory indices in patients with HNSCC receiving anti-PD-1 monotherapy: C-reactive protein (CRP), neutrophil-to-lymphocyte ratio (NLR), platelet-to-lymphocyte ratio (PLR), and the systemic immune inflammation index (SII). A total of 79 patients were included in this retrospective analysis. Optimal cutoff values were determined using receiver operating characteristic curve analysis to stratify patients into high- and low-inflammation groups. Chi-square tests were used to evaluate differences in treatment response. Progression-free survival (PFS) and overall survival (OS) were assessed and compared using Kaplan–Meier analysis and log-rank testing, alongside both univariable and multivariable Cox regression models. Elevated CRP levels were associated with a reduced disease control rate. In univariable analysis, patients in the high-inflammation groups showed significantly worse OS and PFS for all assessed inflammatory indices. In multivariable analysis, CRP and combined positive score remained independently significant predictors of both OS and PFS, while PLR was an independent predictor of OS. These findings suggest that a high level of systemic inflammation is associated with poorer outcomes during anti-PD-1 therapy in HNSCC. Among the evaluated indices, CRP stood out as an independent and clinically useful biomarker, providing a simple, widely available tool that could potentially serve as a practical instrument for clinicians in the management of HNSCC during anti-PD-1 treatment.

## 1. Introduction

Head and neck squamous cell carcinoma (HNSCC) is currently the sixth most common tumor entity worldwide and accounts for nearly 900,000 new cases and 450,000 deaths each year [1]. Curative treatment modalities for locally or locoregionally restricted HNSCC usually consist of integrating surgery, radiotherapy and systemic therapy. However, in the event of disease recurrence or metastasis, prognosis is dismal and therapeutic options are limited. For many years the standard of care for first-line systemic therapy for recurrent or metastatic HNSCC (RM-HNSCC) was the EXTREME regimen consisting of platinum-based chemotherapy, 5-FU and cetuximab, resulting in a median overall survival of less than a year. Programmed Cell Death Protein 1 (PD-1) inhibitors such as Nivolumab and Pembrolizumab in R/M-HNSCC, which were first approved as second-line treatment and have been extended to first-line therapy [2], caused an improved survival with a stable quality of life compared to standard of care. However, the objective response rate remains modest, at around 18% [3]. Moreover, until now it remains unclear which patients exactly are responding to PD-1 inhibitors. To date, mismatch repair status [4] and tumor mutation burden [5] are the only established tumor-agnostic biomarkers for selecting patients for immune checkpoint inhibitor (ICI) therapy in solid tumors. Furthermore, recent data suggests that local inflammation, tumor immune infiltration and tumor programmed death-ligand 1 (PD-L1) expression seem to be associated with response rate and survival in PD-1 inhibition in HNSCC [6]. However, predictive biomarkers for the treatment response are still not available. Interestingly, there is growing evidence that the response to PD-1 inhibition is not only associated with local but also with systemic inflammation.

Many chronic diseases, such as obesity, type-2 diabetes, atherosclerosis, and cancer, are characterized by a state of low-grade systemic inflammation [7]. Particularly in cancer, systemic inflammation is recognized as a major driver of disease development and progression. Evidence indicates that systemic inflammation significantly impacts outcomes in various tumor entities [8], including HNSCC [9]. The systemic inflammatory response involves many organ systems. Still, the usual tools to measure the degree of inflammatory activation in clinical practice are blood-borne markers. These include circulating leukocytes, erythrocyte sedimentation rate, acute-phase proteins such as CRP, haptoglobin, and procalcitonin, and pro-inflammatory cytokines like Interleukin-6. Additionally, there are well-established scoring indices used to estimate the magnitude of inflammation. These include the neutrophil-to-lymphocyte ratio (NLR), the platelet-to-lymphocyte ratio (PLR), and the systemic immune inflammation index (SII). There are numerous data showing the prognostic value of these scores in HNSCC. Specifically, increased systemic inflammation appears to be associated with a worse prognosis in the treatment of both primary [9,10] and recurrent or metastatic HNSCC [11]. However, the predictive role of systemic inflammation in the outcome of HNSCC treated with immune checkpoint inhibition such as PD-1 Inhibitors has not been discovered in detail. Hence, the aim of this study is to evaluate the role of systemic inflammation measured by CRP as well as the NLR, PLR, and SII on response and survival in R/M-HNSCC treated by single-agent Nivolumab and Pembrolizumab therapy.

## 2. Results

### 2.1. Cohort Characteristics

A total of 79 patients were included in the analysis. Among them, 69 were male and 10 female, with a median age of 61 years. Of these patients, 58 were current or former smokers, and 28 had a history of high-risk alcohol consumption as defined by WHO criteria. Regarding treatment, 47 patients received pembrolizumab and 32 received nivolumab. Most patients received immune checkpoint inhibition as a first-line therapy, while 13 were treated in the second-line setting and 3 in the third-line setting. In the majority of cases, therapy was initiated due to R/M-HNSCC. Two patients not in the recurrent setting declined surgery or radiotherapy and instead received primary PD-1 inhibition. The most common indication for therapy was locoregional disease (37 cases, including the two cases treated with primary PD-1 inhibition). Additionally, 28 patients presented with distant metastases without locoregional disease, while 14 had both locoregional and distant recurrence. Combined Positive Score (CPS) values were available for 58 patients, of whom 33 had a CPS ≥ 20. Immune-related adverse events (irAEs) occurred in 18 cases.

Median values were 1.2 mg/dl for CRP (Interquartile range [IQR], 0.5–3), 7.0 for the NLR (IQR, 4.9–12.6), 364 for the PLR (IQR, 240–515) and 1816 for the SII (IQR, 1220–3148). Patients were stratified into “Inflammation High” and “Inflammation Low” groups for each inflammation index, using optimal cut-off points determined by the Youden Index: 2.95 for CRP, 5.9 for the NLR, 376 for the PLR and 1816 for the SII. Baseline characteristics differed between the CRP^Low^ and CRP^High^ groups regarding high-risk alcohol consumption and between the groups for the PLR and SII regarding age. Interestingly, irAEs occurred more often in the inflammation low groups for each index, with significant results for CRP, the NLR and SII (Supplementary Table S1).

### 2.2. Follow-Up and Treatment Response

The median follow-up time was 10 months (IQR, 5–29; range, 2–71), and the median number of treatment cycles administered was six (IQR, 5–12). Across the entire cohort, 8 patients (10%) had a complete response, 17 (21.5%) a partial response, and 7 (9%) stable disease. Progressive disease was observed in 46 patients (58%). Therefore, the overall response rate (ORR) was 31.5% and the disease control rate (DCR) was 42%. Median time to progression was 3.9 months.

Each inflammatory index group was analyzed with respect to the ORR and DCR. No significant differences were observed between groups defined by the NLR, PLR, or SII. However, the disease control rate was significantly worse in the CRP^High^ group compared to the CRP^Low^ group (Table 1).

### 2.3. Survival Analysis

**Figure 1:**
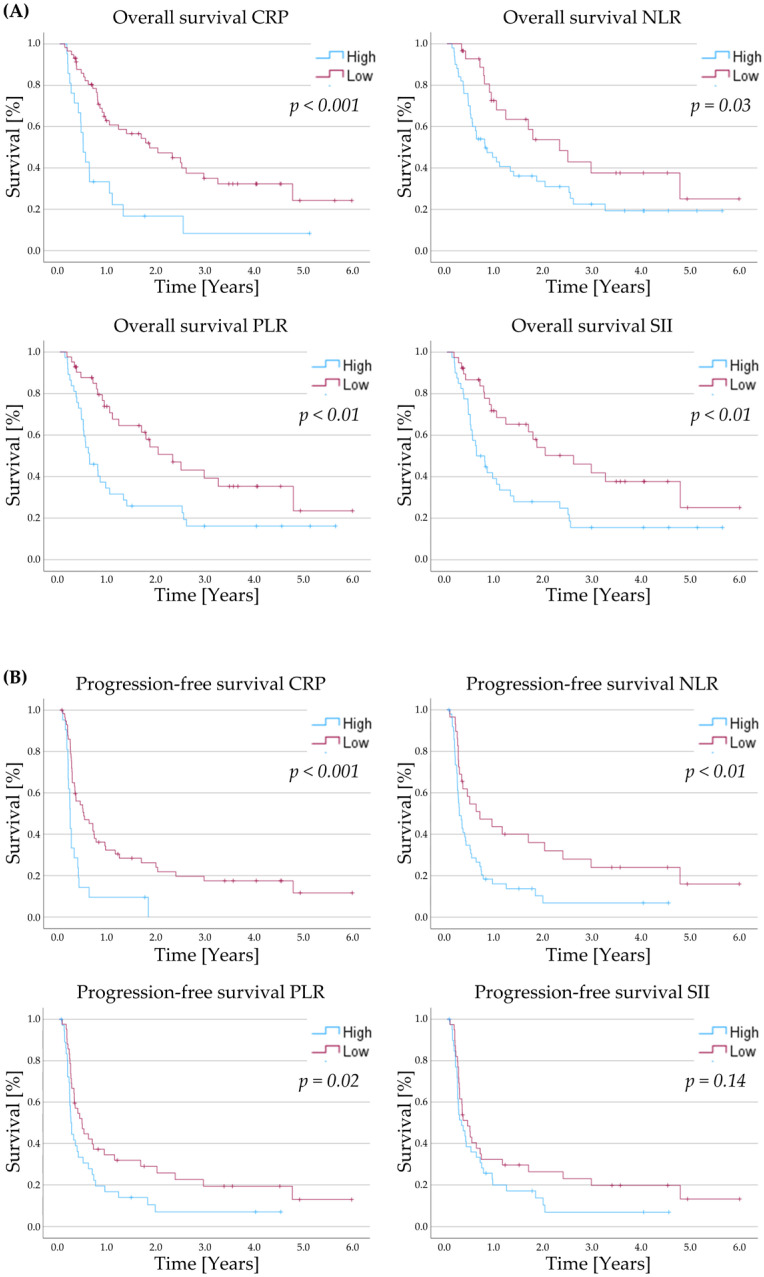
Kaplan–Meier survival curves illustrating (**A**) overall survival and (**B**) progression-free survival, comparing patients with high versus low levels of each inflammation index. C-reactive protein (CRP), neutrophil-to-lymphocyte ratio (NLR), platelet-to-lymphocyte ratio (PLR), and systemic immune inflammation index (SII).

The predictive value of serum inflammatory markers was evaluated in relation to OS. Kaplan–Meier analyses demonstrated that patients with high levels of each inflammatory index had significantly poorer overall survival (Figure 1).

This finding was further supported by univariable Cox regression analysis, which revealed a significantly increased hazard of death among patients with high inflammatory marker levels. In contrast, a CPS > 20 was associated with a significantly reduced hazard of death. In the multivariable analysis, CPS, CRP and the PLR remained independent significant predictors of OS (Table 2).

Similarly, for PFS, Kaplan–Meier curves indicated that higher levels of all inflammatory indices except for the SII were associated with significantly poorer outcomes. In univariable Cox regression, CRP, NLR and PLR retained statistical significance with increased hazards of disease progression for patients with high inflammatory markers, whereas the SII did not. Moreover, both a CPS > 20 and the occurrence of immune-related adverse events (irAEs) were associated with a reduced hazard of disease progression. In the multivariable analysis, CPS and CRP remained independent significant predictors of PFS (Table 3).

## 3. Discussion

The present study evaluated the predictive value of blood-borne inflammation markers including CRP, NLR, PLR, and SII in relation to treatment response and survival in patients with HNSCC receiving anti-PD-1 immunotherapy. Elevated levels of these inflammatory markers were associated with poorer OS and PFS. In addition, high serum CRP levels were significantly associated with a lower DCR.

These findings are also consistent with previous research examining the relationship between systemic inflammation and prognosis in HNSCC patients receiving treatments other than immunotherapy. Several studies have demonstrated that elevated NLR values are associated with poorer outcomes in patients undergoing surgical therapy alone, primary chemoradiotherapy, or surgery followed by adjuvant radiotherapy [9,12,13]. Another blood-based parameter that has gained attention as a potential prognostic biomarker in HNSCC is CRP. Elevated pretreatment serum CRP levels have been associated with poorer clinical outcomes. A meta-analysis of 17 studies involving a total of 4449 patients demonstrated that high CRP levels were significantly associated with reduced OS and PFS in HNSCC [14]. This finding is supported by another study of 208 HNSCC patients, which also identified elevated CRP as an unfavorable prognostic factor for both OS and PFS [15].

Recent studies have increasingly supported the role of systemic inflammation as a predictive factor in ICI therapy for HNSCC. Data from other single-center cohort studies support the finding that a high NLR is a negative predictive parameter for both progression [16,17] and overall survival [17,18] in patients treated with anti-PD-1 immunotherapy, with or without chemotherapy. Matsumura et al. analyzed 65 patients with R/M-HNSCC and found that in univariable analysis, NLR, PLR, and SII were significant negative predictors of PFS and OS during anti-PD-1 immunotherapy. However, none of these markers remained significant in multivariable analysis [19], contrasting with our results, in which PLR was also identified as an independent predictor for PFS. A multicenter study including 119 patients treated with pembrolizumab monotherapy reported that higher NLR and PLR values were associated with significantly shorter OS [20]. Finally, a meta-analysis of 14 studies involving 929 patients demonstrated that an elevated NLR is associated with poorer OS, PFS, treatment response, and disease control, suggesting that NLR is a robust predictive biomarker for outcomes in HNSCC patients treated with immune checkpoint inhibitors [21].

Emerging evidence suggests that CRP may also have a predictive role in ICI therapy for HNSCC. A high CRP level measured during treatment has been identified as an adverse predictive marker for both OS and PFS, indicating that CRP could function not only as a prognostic [14] but also as a predictive biomarker. These findings align with our own results, demonstrating that elevated pretreatment CRP is an independent and significant negative predictor of OS and PFS in HNSCC. Furthermore, this association has been observed across multiple tumor types. A recent study showed that high CRP levels at the initiation of ICI therapy were associated with significantly reduced OS and PFS across various cancer entities [22]. In a meta-analysis including 6124 patients, elevated baseline CRP levels consistently predicted poorer survival outcomes, regardless of cancer type or the specific ICI used [23]. In non-small cell lung cancer (NSCLC), pretreatment CRP was validated as a robust predictor of OS and PFS in both a discovery and an external validation cohort of overall 191 patients [24]. Similarly, in metastatic clear-cell renal cell carcinoma, higher baseline CRP levels were associated with significantly worse OS and PFS in patients treated with nivolumab [25].

Beyond baseline levels, increasing evidence suggests that CRP kinetics may also have predictive value for treatment response and survival in patients receiving ICI. In HNSCC, CRP non-responders exhibited higher rates of progressive disease and poorer OS and PFS compared to CRP responders [16]. Comparable findings have been reported in other tumor types, including NSCLC [26], melanoma [27], and renal cell carcinoma [28]. These observations suggest that not only pretreatment CRP values but also on-treatment kinetics may provide clinically meaningful information. Important limitations persist, as definitions, timing, and thresholds of CRP response vary across studies.

These findings align with observations in other tumor entities, where ongoing systemic inflammation has been associated with poorer outcomes during ICI therapy [22]. Yang et al. showed that in patients with intrahepatic cholangiocarcinoma, high NLR and SII have a negative predictive value for the response to PD-1 inhibition [29]. Several studies demonstrated that the response of NSCLC shows a negative correlation with increased inflammation levels. For example, Peng et al. showed that increased NLR and Diem et al. that increased NLR and PLR values are associated with a lower response to PD-1 inhibition [30,31]. Nardone et al. showed that in NSCLC patients treated with anti PD-1 immunotherapy, elevated PCT, CRP and erythrocyte sedimentation rate indicate a poorer prognosis [32].

The CPS serves as a potential predictive biomarker for response to anti-PD-1 immunotherapy in HNSCC. Here, higher CPS values are generally associated with improved response rates and overall survival [33]. In line with this, this study also demonstrated that a high CPS was significantly associated with an improved OS and PFS in univariable and multivariable analysis, further supporting its value as a predictive marker for ICI response in HNSCC.

This study has several limitations. As this was a single-center retrospective analysis, the generalizability of our findings is limited, as the patient population, treatment practices, and clinical documentation may not fully reflect broader real-world settings. The relatively small sample size (*n* = 79) further restricts the statistical power of our analyses, particularly for subgroup evaluations. This is most evident for CPS, which was only available for 58 patients, thereby reducing the robustness of CPS-related analyses. The retrospective design also introduces potential biases, including selection bias and incomplete or inconsistent documentation. Therefore, the clinical implications of the current findings remain limited. Future multicenter prospective studies with larger and more diverse cohorts will be essential to validate and strengthen these results.

Nevertheless, in line with existing data from both other tumor entities and HNSCC patients, our results support the association between elevated markers of systemic inflammation and poorer outcomes in anti-PD-1 immunotherapy for HNSCC. Systemic inflammation is known to influence tumor progression, metastasis, recurrence, and anti-tumor immunity [34]. Its immunosuppressive effects are largely mediated through the induction of T cell exhaustion. Chronic systemic inflammation has been shown to drive T-cell exhaustion in many chronic infections [35] as well as in cancer [34]. During chronic inflammation, persistent upregulation of PD-1 expression contributes to T cell exhaustion and functional impairment [36]. Given the importance of effective T cell responses for the success of anti-PD-1 immunotherapy, this mechanism could contribute to understanding the observed link between elevated systemic inflammation and less favorable clinical outcomes. However, further research is needed to confirm this association and clarify the underlying pathways.

In summary, this study demonstrates that elevated systemic inflammation is strongly associated with worse outcomes in patients with HNSCC receiving anti-PD-1 immunotherapy, underscoring the relevance of inflammation in this setting. Among the evaluated indices, CRP consistently emerged as the most robust and independent predictor of response and survival. As there is an urgent need for reliable biomarkers to guide treatment response prediction and prognosis in HNSCC, measuring CRP provides a feasible solution. Given its availability in routine clinical practice and its low cost, CRP could potentially serve as a practical tool for clinicians in the management of HNSCC during anti-PD-1 treatment.

## 4. Material and Methods

This retrospective single-center analysis was approved by the local ethical committee (IRB number: 2025-262-dvhd). Patients who were diagnosed with HNSCC and treated with Nivolumab or Pembrolizumab as single-agent therapy between June 2017 and August 2024 were included for analysis. Exclusion criteria were tumor combination therapy with chemotherapy, targeted therapy, or other immune checkpoint inhibitors, unavailable differential blood counts, or a missing clinical or radiological target lesion. Furthermore, patients with hematological disorders or systemic corticosteroid use, which may affect differential blood counts, were excluded from analysis. Seventy-nine patients were identified for further analysis.

Demographics, treatment history, site of tumor recurrence or metastasis, histopathological data, PD-L1 status, blood values, and survival data were obtained from electronic medical records. Tumor staging was based on the 8th Edition of the UICC TNM Classification of Malignant Tumors. The aim of the study was to investigate the predictive value of pretreatment inflammatory markers including C-reactive protein (CRP), neutrophil-to-lymphocyte ratio (NLR), platelet-to-lymphocyte ratio (PLR), and systemic immune inflammation index (SII), on treatment response, progression-free survival (PFS) and overall survival (OS). PFS was calculated as the time between start of PD-1 inhibition and disease progression or death from any cause. Tumor response was either assessed according to the Response Evaluation Criteria in Solid Tumors (RECIST) 1.1 based on follow-up CT scans or clinically in cases of unequivocal tumor progression. OS was calculated as the time between start of PD-1 inhibition and death from any cause. Patients still responding to treatment or still alive on January the 31st 2025 were censored in the survival analysis.

The Combined Positive Score (CPS) was calculated as the number of PD-L1-stained cells (tumor cells, lymphocytes, and macrophages) divided by the total number of viable tumor cells, multiplied by 100.

Differential blood counts were assessed on the day of anti-PD-1 treatment initiation. Systemic inflammation indices were calculated as follows: NLR (neutrophil count divided by lymphocyte count), SII (platelet count multiplied by NLR) and PLR (platelet count divided by lymphocyte count). The overall response rate was used to classify patients into responders and non-responders. A receiver operating characteristic curve was generated to evaluate the diagnostic performance of the blood inflammation indices. To identify the optimal cut-off points that maximized both sensitivity and specificity, the Youden Index was then applied. Based on these cut-off points, the cohort was then stratified into “Inflammation High” and “Inflammation Low” groups for each individual index.

All survival analyses were conducted for PFS and OS. Kaplan–Meier survival curves were generated and compared using the log-rank test. A *p*-value < 0.05 was considered statistically significant. In addition, univariable and multivariable Cox regression analysis were utilized to estimate hazard ratios (HRs) for PFS and OS. Univariable Cox regression analysis was performed for each inflammation index and additional clinically relevant factors. Factors with a *p*-value < 0.05 in the univariable analysis were included as potential confounders in the multivariable analysis. In the multivariable model, factors with *p* < 0.05 were deemed statistically significant and reported along with their corresponding HR and 95% confidence intervals (95% CIs). Chi-squared or Fisher exact testing was used for patient characteristics, ORR and DCR for each inflammatory index. Fisher exact testing was used in cases where at least one cell count was below 5. All statistical analyses were performed using SPSS, version 29.0.0.0 (241) (IBM Corporation, Armonk, NY, USA).

## Figures and Tables

**Table 1:** Treatment response. Patients were stratified into “Inflammation High” and “Inflammation Low” groups by calculated cut-off values for each marker. Treatment response rates were compared between these groups using the Chi-squared test or Fisher’s exact test, as appropriate. C-reactive protein (CRP), complete remission (CR), disease control rate (DCR), neutrophil-to-lymphocyte ratio (NLR), overall response rate (ORR), progressive disease (PD), partial remission (PR), platelet-to-lymphocyte ratio (PLR), stable disease (SD) and systemic immune inflammation index (SII).

Response	CRP ≤ 2.95	CRP > 2.95	*p*-Value	NLR ≤ 5.9	NLR > 5.9	*p*-Value	PLR ≤ 376	PLR > 376	*p*-Value	SII ≤ 1816	SII > 1816	*p*-Value
No. patients	58	21		29	50		43	36		39	40	
Response												
CR	8	1		5	4		7	2		5	4	
PR	14	3		6	11		8	9		7	10	
SD	7	0		5	2		6	1		5	2	
PD	29	17		13	33		22	24		22	24	
ORR			0.18			0.47			0.68			0.69
CR + PR	22	4		11	15		15	11		12	14	
SD + PD	37	17		18	35		28	25		27	26	
DCR			0.02			0.07			0.16			0.75
CR + PR + SD	29	4		16	17		21	12		17	16	
PD	30	17		13	33		22	24		22	24	

**Table 2:** Univariable analysis and multivariable analysis of overall survival. Confidence interval (CI), combined positive score (CPS), C-reactive protein (CRP), hazard ratio (HR), immune-related adverse events (irAEs), neutrophil-to-lymphocyte ratio (NLR), platelet-to-lymphocyte ratio (PLR), and systemic immune inflammation index (SII).

Variables	Univariable Analysis		Multivariable Analysis	
	HR (95% CI)	*p*-Value	HR (95% CI)	*p*-Value
Gender	0.9 (0.38–2.11)	0.8		
Age	1.01 (0.98–1.03)	0.56		
Drug	1.04 (0.6–1.8)	0.89		
irAE	0.71 (0.37–1.36)	0.3		
Smoking	1.45 (0.76–2.77)	0.26		
CPS	0.49 (0.26–0.95)	0.03	0.34 (0.17–0.7)	<0.01
CRP	2.73 (1.52–4.89)	<0.001	2.2 (1.03–4.73)	0.04
NLR	1.9 (1.04–3.47)	0.04	1.1 (0.38–2.9)	0.53
PLR	2.54 (1.46–4.43)	<0.01	2.7 (1.0–7.21)	0.045
SII	2.21 (1.26–389)	<0.01	1.22 (0.46–3.22)	0.7

**Table 3:** Univariable analysis and multivariable analysis of progression-free survival. Confidence interval (CI), combined positive score (CPS), C-reactive protein (CRP), hazard ratio (HR), immune-related adverse events (irAEs), neutrophil-to-lymphocyte ratio (NLR), platelet-to-lymphocyte ratio (PLR), and systemic immune inflammation index (SII).

Variables	Univariable Analysis		Multivariable Analysis	
	HR (95% CI)	*p*-Value	HR (95% CI)	*p*-Value
Gender	1.04 (0.5–2.2)	0.92		
Age	1.0 (0.97–1.0)	0.8		
Drug	0.89 (0.55–1.47)	0.66		
irAE	0.54 (0.29–0.99)	0.04	0.69 (0.36–1.33)	0.4
Smoking	1.18 (0.68–2.06)	0.55		
CPS	0.49 (0.27.0,87)	0.01	0.4 (0.22–0.76)	<0.01
CRP	2.55 (1.48–4.41)	<0.001	2.0 (1.11–3.7)	0.03
NLR	2.05 (1.2–3.49)	<0.01	1.18 (0.55–2.59)	0.38
PLR	1.78 (1.09–2.92)	0.02	1.47 (0.76–2.85)	0.55
SII	1.44 (0.88–2.35)	0.15		

## Data Availability

The data presented in this study are available on request from the corresponding author. The data are not publicly available due to reasons of legal data protection and ethical restrictions.

## Supplementary Materials

The following supporting information can be downloaded at https://www.mdpi.com/article/10.3390/ijms26189154/s1.

## Abbreviations

The following abbreviations are used in this manuscript: Neutrophil-to-lymphocyte ratio (NLR), Platelet-to-lymphocyte ratio (PLR), Confidence interval (CI), C-reactive protein (CRP), hazard ratio (HR), systemic immune inflammation index (SII), Head and neck squamous cell carcinoma (HNSCC), recurrent or metastatic HNSCC (RM-HNSCC), Programmed Cell Death Protein 1 (PD-1), Programmed death-ligand 1 (PD-L1), progression-free survival (PFS), overall survival (OS), Interquartile range (IQR), non-small cell lung cancer (NSCLC), combined positive score (CPS).
