# Supplementary material for: Blood-Based Inflammatory Markers Are Associated with Treatment Outcomes in Head and Neck Squamous Cell Carcinoma Receiving Anti-PD-1 Therapy: CRP as a Superior Predictive Marker
Source: Int J Mol Sci. 2025 Sep 19;26(18):9154. doi: 10.3390/ijms26189154 (PMC12471243; doi:10.3390/ijms26189154)
Supplement: Supplementary file 1 [file ijms-26-09154-s001.zip › ijms-3841186-supplementary.pdf]

Characteristics	N (%)	CRP			NLR			PLR			SII		
		≤ 2.95	> 2.95	*p*-value	≤ 5.9	> 5.9	*p*-value	≤ 376	> 376	*p*-value	≤ 1816	> 1816	*p*-value
Gender n (%)													
Male	69 (87%)	51	18	0.72	25	44	1.0	38	31	0.76	34	35	0.97
Female	10 (13%)	7	3		4	6		5	5		5	5	
Age													
> 65	33 (42%)	24	9	0.91	10	23	0.32	25	12	**0.03**	23	14	**0.03**
< 65	46 (58%)	34	12		19	27		18	24		16	26	
Smoking history, n (%)													
Never smoked	21 (66%)	17	4	0.56	10	11	0.23	12	9	0.77	13	8	0.18
Previous or current	58 (34%)	41	17		19	39		31	27		26	32	
High risk alcohol													
No consumption, n (%)	51 (65%)	20	13	**0.03**	16	35	0.18	26	25	0.41	24	27	0.58
Previous or current	28 (35%)	38	8		13	15		17	11		15	13	
Treatment, n (%)													
Pembrolizumab	47 (59%)	36	11	0.36	14	33	0.21	25	22	0.79	21	26	0.31
Nivolumab	32 (41%)	22	10		15	17		18	14		18	14	
Disease site, n (%)													
Locoregional recurrence	37 (47%)	25	12	0.54	16	21	0.51	21	16	0.84	17	20	0.77
Distant metastases	28 (35%)	22	6		9	19		14	14		14	14	
Locoregional + distant recurrence	14 (18%)	11	3		4	10		8	6		8	6	
Anti-PD-1 treatment line													
First line	63 (80%)	46	17	0.92	21	42	0.37	34	29	0.91	29	34	0.49
Second line	13 (16%)	10	3		6	7		7	6		8	5	
Third line	3 (4%)	2	1		2	1		2	1		2	1	
CPS													
>20	33 (42%)	24	9	0.95	10	23	0.23	19	14	0.9	17	16	0.57
<20	25 (32%)	18	7		19	27		14	11		11	14	
irAE													
yes	18 (23%)	17	1	**0.03**	11	7	**0.01**	13	5	0.08	11	7	**0.04**
no	61 (77%)	41	20		18	43		30	31		17	33	

Supplementary table 1. Patient characteristics. Patients were stratified into "Inflammation High" and "Inflammation Low" groups by calculated cut-off values for each marker. Clinical characteristics were compared between these groups using the Chi-squared test or Fisher's exact test, as appropriate. *Combined positive score (CPS), C-reactive protein (CRP), immune-related adverse events (irAE), neutrophil-to-lymphocyte ratio (NLR), platelet-to-lymphocyte ratio (PLR), and systemic immune-inflammation index (SII)*
